# Experience of nature and times of silence as a resource to cope with the COVID-19 pandemic and their effects on psychological wellbeing—Findings from a continuous cross-sectional survey in Germany

**DOI:** 10.3389/fpubh.2022.1020053

**Published:** 2022-11-07

**Authors:** Arndt Büssing, Daniela Rodrigues Recchia, Klaus Baumann

**Affiliations:** ^1^Professorship Quality of Life, Spirituality and Coping, Faculty of Health, Witten/Herdecke University, Herdecke, Germany; ^2^IUNCTUS - Competence Center for Christian Spirituality, Philosophical-Theological Academy, Münster, Germany; ^3^Department of Research Methods and Statistics in Psychology, Faculty of Health, Witten/Herdecke University, Witten, Germany; ^4^Caritas Science and Christian Social Work, Faculty of Theology, Albert-Ludwig-University, Freiburg, Germany

**Keywords:** perception of nature, times of silence, wondering awe, wellbeing, coping, COVID-19 pandemic

## Abstract

**Background:**

The COVID-19 pandemic with its lockdowns affected social relations and mental health conditions of people worldwide. We aimed to analyze the relevance of nature and times of silence as resources to cope with the pandemic. Of interest were how experiences of nature and times of silence are related to the perception of wondering awe and gratitude and psychological wellbeing and how these have changed during the different phases of the pandemic. Finally, we asked whether *Nature/Silence* would mediate the link between Awe/Gratitude and wellbeing.

**Methods:**

A cross-sectional survey with standardized questionnaires (i.e., PCQ, GrAw-7, BMLSS-10, WHO-5) enrolling participants during the different phases of the COVID-19 pandemic was conducted. The total sample of 5,155 participants from Germany consisted of 65% women and 34% men, with a mean age of 45.0 ± 14.0 years.

**Results:**

Directly after the first lockdown, *Nature/Silence* and Awe/Gratitude scores were high and decreased along with wellbeing with the onset of the second lockdown in winter 2020, while perceived burden constantly increased. *Nature/Silence* was rated lowest by people with reduced wellbeing (eta^2^ = 0.058) and feeling lonely or socially isolated (eta^2^ = 0.042). Predictor analyses revealed that wellbeing as a dependent variable was predicted best by corona-related perception of burden, Awe/Gratitude, reflection of life, and *Nature/Silence* and further by perceived changes in terms of relationships and spirituality (R^2^ = 0.55). In mediation analyses, Awe/Gratitude proved to be a significant predictor for *Nature/Silence* (β = 0.55, p< 0.0001) and wellbeing (β = 0.05, *p* < 0.0001). The mediation analysis explained 37% of the variability in the data. The direct influence of Awe/Gratitude on wellbeing was estimated as β = 0.09 (*p* < 0.0001), and the mediation effect of *Nature/Silence* on the link between Awe/Gratitude and wellbeing was significant, too (β = 0.03, *p* < 0.0001), explaining 25% of the total effect.

**Conclusion:**

*Nature/Silence* and Awe/Gratitude were used as relevant resources during the pandemic, although they cannot fully buffer the negative effects of the social restrictions that resulted in decreases in wellbeing and increases in perceived burden. Perception of nature as a sensitizer of positive experiences particularly during difficult phases of life could be trained to stabilize wellbeing and thus to contribute to public health.

## Introduction

The COVID-19 pandemic has affected the life of people worldwide, resulting in an increase in infection rates with the coronavirus SARS-CoV-2 and subsequent increases in death rates. As a consequence of protecting people at risk, public life was significantly restricted (“lockdown”). The imposed restrictions such as quarantine, social distancing, cancelation of public events, and private gatherings further burdened people who felt socially isolated, lonely, depressed, anxious, and stressed (1–6). Indeed, the global prevalence of major depression and anxiety disorders increased significantly as a result of the pandemic (7). Other effects such as posttraumatic stress disorder, panic attacks, and other psychological impairments have been observed as a result of the restrictions imposed by the pandemic (8).

Nevertheless, during the first phases of the pandemic and lockdown people also made positive experiences such as personal reflections on what is essential in life to perceive nature and relations more consciously, etc. (9–12). Positive health behaviors were reported (13), further an increase in daily physical activities and better dieting (14) and an increase in caring support by friends and family (15). While these perceived changes may contribute to psychological wellbeing, the individual experiences may be more complex. Some may have felt lonely and socially isolated and thus were depressed, while others were better adjusted and had quite good wellbeing (16). How stressors were perceived and which effects these may have on subjective wellbeing can differ, depending on the individual situation, personality, and coping strategies of the affected people. Coping self-efficacy and resilience as resources were identified to mediate the effect of psychological stressors such depression, anxiety, and stress on psychological wellbeing (17) and thus should be integrated in pandemic-related recovery strategies. Resilience as an ability to adapt to adverse situations is a factor that relates to how one is able to cope with stress and its negative effects (18). However, especially during the first lockdown, it was noticeable that resilience of many people was significantly lower than expected (19). Nevertheless, resilience was more pronounced among those who had the opportunity to get outside “in the sunshine” more often, to exercise more, to have more social support, etc. (19). In fact, during the pandemic it was evident that some people can rely on resources such as encounter with nature as a place of quietness and relaxation, beauty and attraction, and enjoying silent times of reflection and contemplation (9–11). This may facilitate perceiving moments of wondering awe in specific situations with subsequent feelings of gratitude in some people (20), while others were less able to use these resources.

### Aims of the study

The aim of this study was to investigate the relevance of nature and times of silence during challenging periods and how these resources might positively improve psychological wellbeing. We focus exclusively on corona-related changes in the perceptions, attitudes, and behaviors related to nature, particularly going outdoors more often and perceiving nature more intensely, and consciously taking more time for silence (“quietness”) and enjoying quiet times of reflection (“respite”). These four variables were condensed to a factor termed *Nature/Silence* (9). Wondering awe is a key aspect to be considered (11) and evaluated together with the positive changes as a buffer to overall wellbeing. We therefore further aimed to analyze whether Awe/Gratitude as a resource has direct effects on psychological wellbeing of participants recruited during the pandemic and whether this effect is mediated by the perception of *Nature/Silence*, which could independently contribute to wellbeing.

### Nature as a resource and mental health

To compensate the social implications of the lockdowns, outdoor spaces, both green spaces (e.g., forests and parks) and blue spaces (e.g., lakes, rivers), and also indoor experiences of nature became more important (21). During the first lockdown in 2020, many used the “extra time” for walks in nature and for their family. Many now had the time to pay more conscious attention to what was usually taken for granted. This renewal of nature-seeking attitudes and behaviors might also have been motivated by the wish to connect with something that is reliable and beauty, distracts from worries, and could thus improve mental and physical health. People from Australia reported benefits from visiting green or blue spaces during the lockdown, including “more respite, connection, and exercise” (22). People from Spain under strict lockdown reported that nature helped them to cope with the restrictions—and their emotional stability was better (23).

Even before the pandemic, the experience of nature proved to be beneficial for wellbeing and mood states (24, 25), to reduce stress (26), and to be associated with prosocial behaviors (27). The beneficial effects of conscious forest walking (shinrin-yoku, “forest bathing”) on stress markers and cortisol levels had been underlined in a meta-analysis by Antonelli et al. (28) already before the pandemic. Even when one would consider placebo effects, this does not argue against the benefit of conscious “time-outs” in nature. A systematic review by de Kaijzer et al. (29) stated that access to and interaction with natural environment can improve mental health and wellbeing—although the findings are often inconsistent. Also, the narrative review of evidence during the corona pandemic by Labib et al. (21) underlined positive correlations between exposure to green and blue spaces and mental health, while the effects of physical health were less congruent. In an urban sample from the USA, negative mental health indicators during the first phase of the pandemic were inversely related to the number of accessible neighborhood parks (30). In citizens of Tokyo, utilization of green space and even the “existence of green window views” positively correlated with self-esteem, life satisfaction, happiness, less loneliness, and decreased levels of depression and anxiety (31). However, it might be that it is not only a matter of accessibility (to green areas), but motivation and emotional attraction to these spaces, how one is “captured” and is able to enjoy the times in nature.

### Moments of quietness and loneliness

The topic of enjoying specific moments of quietness during the pandemic is complex. Those who are able to take such quiet times of seclusion in a self-determined manner may be able to benefit from it, while those who feel socially isolated or lonely may not benefit from times of silence and nature as this can trigger their negative feelings because of heightened awareness. In younger people from the USA, it was shown that a small proportion stated that spending time in nature raised their awareness of being socially isolated (32). Elmer et al. (33) argued that “individuals with depression might be more vulnerable to ‘get stuck' in solitude.” The psychological stability might be the crucial point. Nevertheless, silent times of meditation and contemplation were usually reported to improve self-control and relaxation and to have beneficial effects on depression, anxiety, and psychological stress (34, 35). Further, times of silence are considered as a resource for personal growth and wellbeing (36, 37), and being solo in times of silence within nature can have a remarkable impact as (mentally stable) people can make meaningful and memorable experiences (38). However, it might be that some experiences are more beneficial than other experiences. A study from the UK showed a “greater connectedness to nature and restoration following visits to rural and coastal locations compared with urban green space” (39). Thus, the quality of nature experience seems to be essential, too.

### Awe perceptions and wellbeing

When one is able to stop and consciously perceive a green or blue space of nature as an emotional touching experience and thus to enjoy such times, one may also perceive gratitude for what is around us (20). This pausing in wondering awe is triggered by the experience of nature *per se*, by the perception of the sacred in nature, and by unique moments (i.e., times of silence, perception that the given moment is extraordinary, moments of insight and understanding, etc.) (20). From a theoretical point of view, one may differentiate the experience of wondering from deeper feelings of awe, which is an immediate experience that may motivate more reflective experiences (40). Further, the respective perception of wondering awe may rather be an esthetic fascination instead of a profound and intense experience that may even change life (41, 42). Perceptions of awe are reported to be associated with greater wellbeing (20, 43), positive emotions, and less anxiety (44) and to buffer negative feelings (45, 46). Awe/Gratitude was the best predictor of positive changes in perceptions and attitudes due to the COVID-19 pandemic (11, 47). During the pandemic, Awe/Gratitude may have sensitized people to “perceive the world around (including nature and concrete persons) more intensely, probably in terms of, or similar to, posttraumatic growth” (47).

## Materials and methods

### Participants

To investigate the relevance of nature and times of silence as resources to cope with the COVID-19 pandemic in participants from Germany, we conducted a cross-sectional survey with standardized questionnaires. The data were collected through a snowball sampling *via* social networks (e.g., Facebook), websites, and emails that were encouraged to be forwarded to spread the information. The recruitment period was from June 2020 to May 2022. The dataset has 5,155 participants from eight consecutive cohorts within the pandemic: (1) in June 2020 after the first lockdown, (2) between July and September 2020 (summer drop of infection rates), (3) between October 2020 and January 2021 (second wave), (4) in February 2021 (short drop of infection rates), (5) between March and May 2021 (third wave), (6) between June and July 2021 (summer drop of infection rates), (7) between August and November 2021 (fourth wave), and (8) between December 2021 and May 2022 (fifth wave).

Participants were informed about the goal of the study and assured anonymity as well as confidentiality. Any kind of personal identification process, such as IP address, was not recorded, thus following the federal instruction for Data Protection (Bundesdatenschutzgesetzt). By taking part on the study, interested people consented to participate in this study.

As not all participants completed the questionnaire, only those who filled the relevant parts of the questionnaire were regarded as “completers.” For the variables containing missing single values, a multivariate imputation technique was applied so that the analysis can be performed with the full (imputed) dataset.

### Measures

Apart from basic sociodemographic data, participants responded to standardized measures that are described in the following.

#### Perceptions of changes (PCQ) and the *Nature/Silence* subscale

To assess which changes in attitudes, perceptions, and behaviors due to the corona pandemic were perceived by the participants, we used the 32-item *Perception of Change Questionnaire* (PCQ), which has good psychometric properties (Cronbach's alpha = 0.91) (10). The instrument differentiates five main factors: (1) *Nature/Silence*/Contemplation (seven items, Cronbach's alpha = 0.87); (2) Spirituality (five items, Cronbach's alpha = 0.83); (3) Relationships (six items, Cronbach's alpha = 0.80); (4) Reflections on life (three items, Cronbach's alpha = 0.74); and (5) Digital media usage (three items, Cronbach's alpha = 0.74), and an additional three-item factor termed Restrictions (Cronbach's alpha = 0.78) (10).

In PCQ's first version (12 items) applied in tumor patients, the first factor addressing nature and times of silence used four items only (9): “I go outdoors much more often”; “I perceive nature more intensely”; “I consciously take more time for silence”; and “I enjoy quiet times of reflection.” However, the extended version of the PCQ (32 items) was applied in a more general population (10) where three additional items loaded on this factor: “I'm more relaxed than before”; “I come to deal more with myself again”; and “I pay more attention to what's really important in life.” As these three items have shifted the focus of the factor's primary intention, we will use the four-item factor *Nature/Silence* in this evaluation. It has good internal consistency in tumor patients (Cronbach's alpha = 0.82) (9) and also in this more general sample (Cronbach's alpha = 0.87).

The items were introduced by the phrase “Due to the current situation…,” which referred to the COVID-19 pandemic. Representative items of the other scales are as follows: “I pay more attention to what's really important in life,” “I perceive the relationship with my partner/family more intensely,” “I am more concerned about the meaning and purpose of my life,” and “I have confidence in a higher power that supports me.” Agreement or disagreement was scored on a five-point scale (0—does not apply at all; 1—does not truly apply; 2—neither yes nor no; 3—applies quite a bit; 4—applies very much). Each factor is a mean score multiplied by 25 to sum up to 100.

#### Wellbeing index (WHO-5)

The *World Health Organization Five Wellbeing Index* (WHO-5) is a short screening instrument to measure one's current mental wellbeing state (48). The proposed instrument was validated in several studies that underlined its good construct validity (49). Negatively phrased questions as “I have felt cheerful and in good spirits” or positively phrased questions as “My daily life has been filled with things that interest me” are covered. Respondents assess how often they had the respective feelings within the last 2 weeks, ranging from at no time (0) to all of the times (5). The total sum scores ranging from 0 to 25 are reported. Scores < 13 would indicate depressive states.

#### Life satisfaction (BMLSS-10)

To measure overall life satisfaction, the *Brief Multidimensional Life Satisfaction Scale* (BMLSS) was used (50). This 10-item questionnaire addresses the following five main dimensions of life satisfaction: intrinsic (oneself, life in general), social (friendships, family life), external (work situation, habitation), prospective (financial situation, future prospects), and health (current health situation, abilities to deal with daily life concerns). The items can be scored from 0 (very unsatisfied) to 6 (very satisfied).

#### Awe and gratitude (GrAw-7)

To address the frequency of situations where participants experience times of pausing for wondering awe in specific situations (mainly in the nature) with subsequent feelings of gratitude, the seven-item Awe/Gratitude (GrAw-7) scale was applied (51). This scale has good psychometric properties (Cronbach's alpha = 0.82), is not contaminated with specific religious or spiritual terminology, and is applicable also to non-religious people. It uses items such as “I stop and am captivated by the beauty of nature,” “I pause and stay spellbound at the moment,” and “In certain places, I become very quiet and devout.” The items are scored on a four-point Likert scale (0—never; 1—seldom; 2—often; 3—regularly) and were then referred to a 100-point scale.

#### Perceptions of burden

To measure negative perceptions due to the restrictions of the pandemic, a set of five questions was presented, e.g., perception of being: (1) restricted in daily life, (2) under pressure/stressed, (3) fearful and insecure, (4) lonely and socially isolated, and (5) burdened in the financial and economic situation. Answers were measured with five numeric analog scales (NRS), ranging from 0 (not at all) to 100 (very strong). These five variables were combined to a factor labeled “corona-related stressors” (5NRS) with good internal consistency (10).

#### Health behaviors

The frequency of current health practices such as sporting activities and walking in nature and spiritual practices such as meditation and praying was measured with a four-grade scale varying from “never” to “at least once a day”.

### Statistical analysis

We performed descriptive statistical analyses for the sociodemographic variables. Group comparisons are reported with *p*-values and effect sizes for better contextualization of results. Here, eta^2^ values < 0.06 are considered as a small effect, between 0.06 and 0.14 as a moderate effect, and > 0.14 as a strong effect. Cohen's d values < 0.20 are considered as small, between 0.20 and 0.50 as moderate, and > 0.50 as strong effects. For correlation analysis, we chose the Spearman rho coefficient, as it is more robust to skewed data. Here, r values < 0.30 are regarded as small, between 0.30 and 0.50 as moderate, and > 0.50 as strong correlations.

The linear regression modeling with stepwise variable selection was used to evaluate the relationship between the dependent variables wellbeing (WHO-5) and *Nature/Silence* (PCQ) in two separated models. Standardized beta coefficients are presented for model interpretation.

To investigate whether the variable *Nature/Silence* mediates the relationship between Awe/Gratitude (GrAw) and Wellbeing (WHO-5), we applied a mediation analysis (52). In this procedure, both the direct effect from *Nature/Silence* and Awe/Gratitude on wellbeing and the mediation effect are evaluated.

All statistical analyses were performed with the software SPSS 28.0.

## Results

### Description of the sample

The total sample (*n* = 5,155) consisted of 65% women and 34% men, with a mean age of 45.0 ± 14.0 years (Table 1). 25.9% were recruited in June 2020 after the first lockdown, 16.0% between July and September 2020 (summer drop of infection rates), 12.1% between October 2020 and January 2021 (second wave), 4.8% in February 2021 (short drop of infection rates), 10.1% between March and May 2021 (third wave), 2.2% between June and July 2021 (summer drop of infection rates), 20.1% between August and November 2021 (fourth wave), and 8.8% between December 2021 and May 2022 (fifth wave).

**Table 1:** Description of 5,144 participants (from June 2020 to May 2021).

	*N* ^*^	**%**	**Mean ±SD**
**Gender**	5,132	100.0	
Women	3,338	65.0	
Men	1,761	34.3	
Diverse	33	0.6	
**Age groups**	5,058	100.0	
< 30 years	933	18.4	
30–40 years	986	19.5	
41–50 years	1,154	22.8	
51–60 years	1,358	26.8	
>60 years	627	12.4	
**Mean age [years]**	5,055		45.0 ± 14.0
**Partner status**	5,144	100.0	
Single	1,082	21.0	
**Area of profession** ^**^			
Management/administration	684	13.3	
Economy	759	14.8	
Health	916	17.8	
Education	414	8.0	
Handcraft / Trading	291	5.7	
Church / Theology	376	7.3	
Pensioners	107	2.1	
Other	1,797	35.0	
**Religious affiliation**	5,122	100.0	
Catholics	1,875	36.6	
Protestants	1,124	21.9	
Free church/Evangelical	127	2.5	
Other	217	4.2	
None	1,779	34.7	
**Faith as hold in difficult times**	5,064	100.0	
Disagreement	2,139	42.2	
Undecided	1,399	27.6	
Agreement	1,526	30.1	
**Frequency of spiritual practices**			
Praying [0–3]	4,622		1.08 ± 1.27
Meditation [0–3]	4,630		0.80 ± 1.09
**Frequency of health behaviors**			
Walking in nature [0–3]	4,834		1.97 ± 0.84
Sporting activities [0–3]	4,808		1.56 ± 1.00
**Cohorts within the pandemic**	5,144	100.0	
June 2020 (after first lockdown)	1,333	25.9	
July to September 2020 (summer drop)	823	16.0	
October 2020 to January 2021 (second wave)	622	12.1	
February 2021 (short drop)	249	4.8	
March to May 2021 (third wave)	519	10.1	
June to July 2021 (summer drop)	113	2.2	
August to November 2021 (fourth wave)	1,032	20.1	
December 2021 to May 2022 (fifth wave)	453	8.8	
**Quality of life indicators**			
Wellbeing (WHO-5) [0–100]	5,144		49.3 ± 26.2
Corona-related stressors (5NRS) [0–100]	5,144		42.2 ± 25.5

* Some participants did not state sociodemographic data, and thus, % refers to responding persons.

** In some cases, several areas of profession were stated and the number is higher than the absolute number of participants.

Most were living with a partner and 21% as singles. People with a Christian denomination were the majority (36.6% Catholics, 21.9% Protestants, and 3% Free Church members), while 34.7% stated no religious affiliation (Table 1). However, only 30.1% would agree that their faith is a strong hold in difficult times. The overall wellbeing score (WHO-5 100% score) is 49.3 ± 26.2 and that for corona-related stressors (5NRS) 42.2 ± 25.5 (Table 1).

### Experience of nature and times of silence and their relation to perceived changes because of the pandemic and quality of life indicators

Going outdoors and walking in nature (Table 1) was practiced by 27.5% at a daily level, 48.1% at least once per week, 18.5% at least once per month, and never by 6.0%. Regarding the aspects of being in nature and conscientiously taking time for silence, 42.7% of participants declared to go outdoors more often than before, 43.3% perceived nature more intensely than before, 26.2% took consciously more time for silence, and 29.7% enjoyed quiet times of reflection (Table 2).

**Table 2:** Frequency distribution for the aspects of *Nature/Silence*.

		**Does not apply at all (%)**	**Does not really apply (%)**	**Neither yes nor no (%)**	**Applies quite well (%)**	**Definitely applies (%)**	**Mean ±SD [0–4]**
C10 Mindfulness	I go outdoors much more often.	9.7	12.9	34.7	27.4	15.3	2.26 ± 1.16
C11 Mindfulness	I perceive nature more intensely.	9.6	11.3	35.9	29.1	14.2	2.27 ± 1.13
C12 Silence	I consciously take more time for silence.	17.4	16.9	39.5	18.4	7.8	1.28 ± 1.15
C13 Silence	I enjoy quiet times of reflection.	19.3	14.8	36.3	20.4	9.3	1.85 ± 1.21

The combined factor *Nature/Silence* was rated lowest by people with low wellbeing (*p* < 0.0001; eta^2^ = 0.058) and perception of loneliness/social isolation (*p* < 0.0001; eta^2^ = 0.042) (Table 3). Gender-related differences were marginal only (Cohen's d = 0.09), while age had a relevant influence (eta^2^ =0.045) (Table 3).

**Table 3:** Frequency distribution with group comparisons for sociodemographic variables.

		***Nature/Silence***	
	**N**	**Mean**	**SD**
All participants	5,144	51.30	24.60
**Gender**			
Female	3,338	52.14	25.00
Male	1,761	49.86	23.69
Diverse	33	46.02	29.18
F value		5.72	
p value		0.003	
Eta^2^		0.00	
Cohen's d (f vs. m)		0.09	
**Age categories**			
< 21 years	188	41.62	23.24
21–30 years	745	45.96	25.81
31–40 years	986	45.73	23.60
41–50 years	1,154	51.75	23.90
51–60 years	1,358	55.61	23.88
61–70 years	489	58.14	22.93
> 70 years	138	62.68	22.30
F value		39.34	
p-value		<0.0001	
Eta^2^		0.045	
Cohen's d (< 21 y vs. < 70 y)		0.92	
**Wellbeing (WHO-5)**			
Low (<13)	2,503	45.40	24.57
Moderate (13–18)	1,504	55.07	22.70
High (> 18)	1,137	59.30	23.84
F value		158.95	
*p*-value		<0.0001	
Eta^2^		0.058	
Cohen's d (low vs. high)		0.57	
**Loneliness (NRS)**			
Low (0)	1,253	55.48	24.62
Moderate (10–50)	1,950	55.51	22.27
High (> 50)	1,475	44.60	25.76
F value		103.73	
*p*-value		<0.0001	
Eta^2^		0.042	
Cohen's d (low vs. high)		0.43	

Effect size eta^2^ < 0.06 small, 0.06 to 0.14 moderate, > 0.14 strong effects.

Effect size Cohen's d < 0.20 small, 0.20 to 0.50 moderate, >0.50 strong effects.

As shown in Table 4, correlation analyses revealed that *Nature/Silence* was moderately related to Awe/Gratitude, to perceived changes in terms of Spirituality and Reflections on life, and strongly related to Relationships, while it was weakly related to psychological wellbeing and to life satisfaction and inversely to corona-related stressors. In contrast, Awe/Gratitude was moderately related to wellbeing, life satisfaction, and *Nature/Silence* and further to frequency of meditation and praying. The frequency of walking in nature was best related to *Nature/Silence* (r = 0.30) and marginally or weakly only to the other perceived changes, indicators of wellbeing, or health behaviors (Table 4). In consequence, one may assume interaction effects.

**Table 4:** Correlation analyses.

	**Nature / Silence (PCQ)**	**Awe / Gratitude (GrAw−7)**	**Walking in nature**
*Nature/Silence* (PCQ)	1.000	0.421^**^	0.302^**^
Spirituality (PCQ)	0.490^**^	0.455^**^	0.141^**^
Relationships (PCQ)	0.512^**^	0.362^**^	0.174^**^
Reflections (PCQ)	0.324^**^	0.183^**^	0.047^**^
Digital Media usage (PCQ)	0.276^**^	0.181^**^	0.088^**^
Restrictions (PCQ)	−0.266^**^	−0.246^**^	−0.088^**^
Corona-related stressors (5NRS)	−0.213^**^	−0.219^**^	−0.086^**^
Wellbeing (WHO-5)	0.292^**^	0.347^**^	0.160^**^
Life satisfaction (BMLSS-10)	0.224^**^	0.345^**^	0.125^**^
Awe/Gratitude (GrAw-7)	0.421^**^	1.000	0.273^**^
Sporting activities	0.212^**^	0.201^**^	0.280^**^
Walking in nature	0.302^**^	0.273^**^	1.000
Meditation	0.377^**^	0.438^**^	0.212^**^
Praying	0.295^**^	0.401^**^	0.114^**^

** p < 0.0001 (Spearman's rho); moderate (yellow) and strong (orange) correlations are highlighted.

### Courses of *Nature/Silence*, Awe/Gratitude, wellbeing, and stressors during the phases of the pandemic

**Figure 1:**
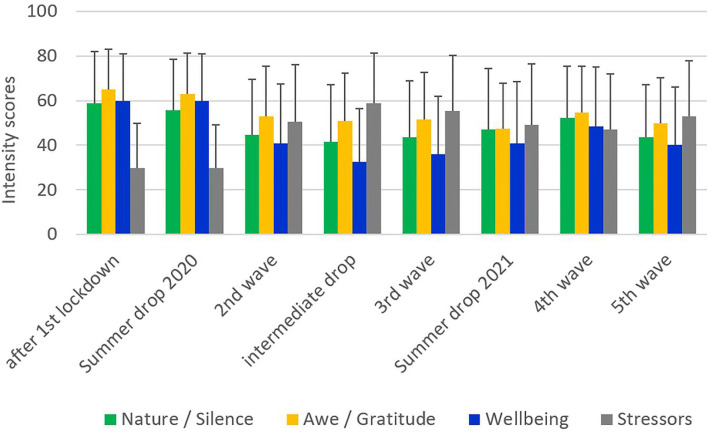
Scores distributions in cohorts within pandemic.

As the resources *Nature/Silence* and perceptions of Awe/Gratitude were moderately positively related (r = 0.42) and the quality of life indicators wellbeing and corona-related stressors were strongly inversely related (r = −0.69), with significant associations between both groups of variables, we analyzed their specific courses during the different phases of the pandemic. As shown in Figure 1, *Nature/Silence*, Awe/Gratitude, and wellbeing scored highest during the first phase of the pandemic in 2020 and decreased with the onset of the second lockdown in 2020 (Figure 1). During the summer drop 2021 and wave 4 in 2021, they started to raise again. Actually, during the later phases of the pandemic both resources (*Nature/Silence* and Awe/Gratitude) scored lower than in the first phase of the pandemic. In contrast, the corona-related stressors increased after the second lockdown in 2020 and remained high.

### Predictors of *Nature/Silence* and wellbeing

As there were several variables with a significant impact on the resource variable *Nature/Silence*, we performed stepwise regression analyses to identify the best predictors. As shown in Table 5, *Nature/Silence* as a dependent variable was explained best by positively perceived Relationships (which already explained 30% of variance), Spirituality, influences of walking in nature, Reflections of life, and wellbeing (which together added further 18% of explained variance). Awe/Gratitude and meditation practices had additional but much weaker positive influences, while corona-related stressors, praying, and life satisfaction had negative influences. These 10 variables together explain 50% of variance. In this model, social isolation had no significant independent effect on *Nature/Silence*.

**Table 5:** Predictors of *Nature/Silence* as a dependent variable (stepwise regression analyses).

**Dependent variable: *Nature/Silence* (PCQ)** **Model 10: F = 451.6, *p* < 0.0001; R^2^ = 0.50**	**Beta**	**T**	***p***
(Constant)		−2.744	0.006
Relationships (PCQ)	0.316	25.487	<0.0001
Spirituality (PCQ)	0.207	12.342	<0.0001
Walking in nature	0.167	15.007	<0.0001
Reflection of life (PCQ)	0.214	16.698	<0.0001
Wellbeing (WHO−5)	0.124	7.437	<0.0001
Meditation	0.073	5.684	<0.0001
Corona–related stressors (5NRS)	−0.093	−6.065	<0.0001
Awe/Gratitude (GrAw−7)	0.073	5.288	<0.0001
Praying	−0.064	−4.419	<0.0001
Life satisfaction (BMLSS−10)	−0.032	−2.067	0.039

Not significant in the model: social isolation.

Wellbeing was predicted best by low perception of corona-related stressors (which alone explained 48% of variance) and inversely by Reflection of life, positively by Awe/Gratitude, and further by perceived changes in Relationships and Spirituality (Table 6). These six variables together explain 55% of variance. In this model, walking in nature, meditation, praying, and social isolation had no significant independent effect in this regression model.

**Table 6:** Predictors of wellbeing as a dependent variable (stepwise regression analyses).

**Dependent variable: Wellbeing (WHO-)** **Model 6: F = 929.8, *p* < 0.0001; R^2^ = 0.55**	**Beta**	**T**	***p***
(constant)		50.771	<0.0001
Corona-related stressors (5NRS)	−0.563	−50.014	<0.0001
Awe/Gratitude (GrAw-7)	0.170	14.119	<0.0001
Reflection of Life (PCQ)	−0.175	−14.582	<0.0001
*Nature/Silence* (PCQ)	0.108	7.998	<0.0001
Relationships (PCQ)	0.077	6.341	<0.0001
Spirituality (PCQ)	0.034	2.736	0.006

Not significant in the model: walking in nature, meditation, praying, and social isolation.

### *Nature/Silence* as mediator between Awe/Gratitude and wellbeing

As described above, we suggested interaction effects between these three variables with *Nature/Silence* as the putative mediator of the link between Awe/Gratitude and wellbeing. In fact, Awe/Gratitude is a significant predictor of *Nature/Silence* (β = 0.55, *p* < 0.0001) which in turn is also a relevant predictor for wellbeing (β = 0.05, *p* < 0.0001). The mediation analysis explained 37% of the variability in the data (R^2^ = 0.37).

**Figure 2:**
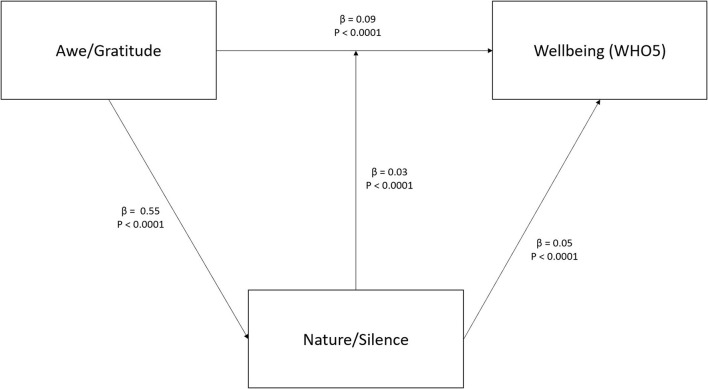
Mediation analysis with *Nature/Silence* as mediator between Awe/Gratitude and wellbeing. Depicted are standardized beta values with p-values.

The direct influence from Awe/Gratitude on wellbeing is estimated as β = 0.09 (*p* < 0.0001), and the mediation effect of *Nature/Silence* on the relationship between Awe/Gratitude and wellbeing is statistically significant (β = 0.03, *p* < 0.0001) and responsible for 25% of the explained total effect of Awe/Gratitude on wellbeing. The total effect from the mediation analysis on wellbeing is estimated as β = 0.12 (*p* < 0.0001). Figure 2 displays the path model for the mediation analysis.

## Discussion

During the pandemic, a large fraction of participants in our survey from Germany used the resources of nature and, a bit less intensively, reflective times of silence (often in nature). The resource (*Nature/Silence*) was declining during the course of the pandemic along with the increase in corona-related burden (stressors) and decline in psychological wellbeing. While the perceived burden remained high during the pandemic, the wellbeing scores improved to a still lower level than before. In contrast, both interconnected resource variables (*Nature/Silence* and Awe/Gratitude) changed largely parallel to each other. The recovery of both resource variables during the fourth wave was related to an improvement in wellbeing and a decline in corona-related burden.

It became obvious that the resource *Nature/Silence* was less relevant for people with low psychological wellbeing (“depressive mood states”) and high perception of loneliness/social isolation and also for younger people. In line with the age effect for this resource, the COVID-19 Mental Disorders Collaborators (7) have approved that particularly the younger generation suffered more from the pandemic-related restrictions with increases in depressive mood states and anxiety than the older generation. In our sample, psychological wellbeing (eta^2^ = 0.068, *p* < 0.001), life satisfaction (eta^2^ = 0.030, *p* < 0.001), and Awe/Gratitude (eta^2^ = 0.053, p<0.001) were significantly lower and perceived burden (eta^2^ = 0.076, *p* < 0.001) was significantly higher in the younger participants as compared to older people. The younger participants seemed to be more at risk of lower wellbeing during the pandemic. They have been less able to perceive nature as a resource of rest and recreation (to distance themselves from their worries) and to be mindfully aware of unique and touching moments in their daily lives, which seem more stressful than in older people. Older people either have learned to distance themselves more from their fears and worries, or have other coping strategies to seize on, and of course, they either have or take more time for such activities to improve their health condition. However, in younger people from the USA aged 14–24 years and recruited in the first phase of the pandemic, 52% stated that spending time in nature made them feel calm, while at least 22% said that it improved their perception of stress and anxiety (32). This means that also younger generations are receptive toward beneficial effects of being out in nature. On the contrary, older people and younger ones differ in how they emotionally react to outdoor environments (53). These environments may trigger positive emotions in older people as they may remember specific situations from life and thereby emotionally connect with their loved ones (53). In qualitative interviews with people >65 years of age from Vancouver, it was found that such places in nature “represented important spiritual and restorative landscapes that promoted feelings of contemplation, spiritual peace, and rejuvenation for participants” (53).

Of course, experience of nature requires access to esthetically touching places and locations and the motivation to go out for walking. During the pandemic, in our sample 27.5% were out for walking every day and 48.1% at least once per week—and 24.5% less often. However, the frequency of being out in nature may not be enough as the attraction and emotional response could be more relevant. In fact, while *Nature/Silence* is moderately interrelated with walking in nature and with perceiving moments of wondering awe and gratitude, their correlation patterns are different. Awe/Gratitude is much stronger related to wellbeing and life satisfaction than *Nature/Silence*, while the frequency of walking in nature is only marginally related to both quality of life indicators. Further, Awe/Gratitude and *Nature/Silence* are moderately related to spiritual practices such as meditation and praying, while walking in nature is much weaker related to both practices. This means that those who are practicing meditation or are praying might be more sensitive toward moments of awe and may value the beauty of nature more—and they are better “trained” in enjoying quiet times in silence, which is an intrinsic part of the meditation practice.

Further, both resources (Awe/Gratitude and *Nature/Silence*) are at least weakly negatively related to corona-related burden, while walking in nature is not relevantly associated with this burden. This means that how often one is approaching nature is not that relevant to buffer the underling stressors; instead, it is of relevance what one perceives while being in nature. The conscious moments of wondering awareness and of being attracted by nature's beauty and by what is emotionally touching and perceived as unique (or sacred) are relevant. One may call it the “special of the inconspicuous,” as something that comes anew into awareness. Nevertheless, both resources are buffers that contribute to wellbeing to some extent. The best predictor of wellbeing during the pandemic was low perception of corona-related burden (or stressors), which alone explained 48% of variance. Nevertheless, Awe/Gratitude was among the further significant contributors (which alone would predict 14% of variance), while *Nature/Silence* alone would predict only 1% of variance in the wellbeing scores.

Nevertheless, this perception of *Nature/Silence* remains an interesting resource that has its own value. It was predicted best by positively perceived changes in Relationships (which explained alone 30% of variance), further by Reflections on life, by changes in terms of Spirituality, by walking in nature, and finally by psychological wellbeing (which together added further 18% of explained variance). This means that being more aware of the relationships (e.g., perception of more intense relationship with partner and family, which became important to feel “safe and at home,” and thus the intention to take more time for them) goes along with becoming more aware of nature as a resource and enjoying quiet times of reflection (e.g., considering meaning and purpose of life and of the lifetime one has, but also perceiving times of loneliness more intensely). Here, the aspect of *intensity of awareness* of all that what was usually taken for granted is central. Walking in nature might be a precondition or facilitator of such perceptions, but it is the ability or openness to resonate with the special moments that is crucial. A person's spirituality and related practices (e.g., meditation or praying) may sensitize for this conscious awareness. This connects this topic to the rich field of mindfulness interventions, which were shown to reduce stress, depression, and anxiety and to improve self-compassion (54–56), thus stabilizing mental health.

Presupposing that touching experiences (in nature, during spiritual practices, etc.) trigger perceptions of awe with subsequent feelings of gratitude (20) and thus contribute to a person's wellbeing, it is consequent to assume a mediation effect of *Nature/Silence* on the link between Awe/Gratitude and wellbeing. In fact, we were able to confirm this mediation effect. The mediation effect of *Nature/Silence* on the relationship between both variables is significant and responsible for 25% of the explained total effect of Awe/Gratitude on wellbeing. This means that the experiential aspect is of relevance: It makes a difference if a person is able to enjoy times of silence while being out in nature and to be perceptive of pausing in wondering awe. These abilities contribute to wellbeing. An interesting mode of action was suggested by Pang and Ruch (57) who pointed to the fact that specific character strengths are related to mindful awareness, i.e., creativity, curiosity, open-mindedness, forgiveness, appreciation of beauty, gratitude, hope, and spirituality, and that mindfulness training may help to cultivate these. In their study, mindfulness training improved participants' appreciation of beauty, love, gratitude, and spirituality (57). This would fit to our findings that not all people may experience the same resources similarly (here, nature and times of silence) and that spiritual practices such as meditation and praying are positively associated with *Nature/Silence* and Awe/Gratitude.

### Limitations

The data were derived from an online survey which was continuously responded to during the different phases of the pandemic. We had no control who was responding and who was not reached by the snowball sampling method. Thus, despite the large number of participants, the findings cannot be claimed to be representative of Germany's society, as the recruitment process may have favored persons with internet access and academic contexts. Obviously, while this selection bias is acceptable to address the research questions, it would be interesting to analyze persons from other contexts and to get a representative picture.

Due to the cross-sectional design of the study, no causal conclusions can be drawn. To account for this, we added data from different recruitment months (resulting in different cohorts). Data from these cohorts indicate that the decrease in wellbeing, Awe/Gratitude, and *Nature/Silence* goes parallel with the increase in perceived burden. As awe and nature perceptions were not increasing during the later courses of the pandemic, we cannot assume “spring time effects” (where walks in nature might be more impressive than during winter times). In fact, *Nature/Silence* and Awe/Gratitude were higher in the fourth wave of the pandemic which started in the second half of 2021, but were nevertheless lower than at the start of the study.

In this study, we differentiated neither participants' economic situation nor their living conditions as urban or rural nor the place they access for their walks in nature, either green or blue areas. With the knowledge that *Nature/Silence* is a mediator for the axis Awe/Gratitude and psychological wellbeing, future studies could further clarify these additional influences.

We have no data how long these visits in nature or times of silence lasted. However, we do not assume that the duration is of outstanding relevance, but the qualitative time.

As our intention was to assess participants' wellbeing (and to nevertheless get hints of states of low wellbeing) but not to diagnose depression, we used the WHO-5 as a screener instead of established “diagnostic” instruments such as the Beck Depression Inventory (BDI) or the Hospital Anxiety and Depression Scale (HADS). The WHO-5 and the BDI-II were both identified as acceptable screening instruments, where the WHO-5 was appreciated stronger of mood screening (58). The WHO-5 is further recommended as a first-step screening instrument, while the BDI is the second step of the screening procedure (59).

### Conclusion

The perception of nature as a resource of respite and re-connection and the ability to enjoy quite times of reflection (“Silence”) on the one hand and pausing in wondering awe (which is most often experienced in nature) with subsequent feelings of gratitude on the other hand were utilized as stabilizing resources during the pandemic. While they cannot fully buffer the negative effects of the social restrictions, they nevertheless contribute to psychological wellbeing. Awe/Gratitude as an ability to perceive the sacred in one's life (whatever is regarded as sacred by the individual persons) even during the pandemic was related to wellbeing, and this effect was mediated by the perception of nature and reflective times of silence.

However, when it is true that people who feel lonely because of the pandemic restrictions and miss their social contacts (and thus feel emotionally burdened) are less able to enjoy quiet moments in nature and are less able to stand in wondering awe—how can they be supported? Of course, it is true that depressive states (which can be both the cause and the result of loneliness) impair people's attention and cognitive control (60) and thus prevent them from extraordinary experiences and decrease their motivation. It seems that low-threshold opportunities for positive experiences are needed, particularly in urban areas with restricted availability of esthetically attractive green spaces. This is a challenge for urban planners. Perception of nature in green and blue areas as a sensitizer of positive experiences, particularly during difficult phases of life, could be facilitated to stabilize people's wellbeing and would thus contribute to public health. Supportive intervention programs are needed that could be used also during future pandemic measures with their social restrictions to minimize the negative impacts and thus to stabilize public mental health. A simple, low-threshold intervention to sensitize awareness for specific moments of wondering awe during daily life is currently under development.

The findings of this study could inform public health policies. Their aim should be to support the broad spectrum of people living in heterogenic societies, with a specific focus on those who feel lonely and socially isolated. The crucial point is how these can be encouraged to spend more time outside in green and blue spaces and to facilitate positive experiences in nature that may help to stabilize their health and wellbeing. Urban planners should be aware that these areas are a cost-effective resource that contribute to peoples' quality of life and may help to decrease risk factors for the development of mental health affections during the pandemic to some extent. However, as in general particularly elderly suffer from social isolation and may be physically limited to access green areas that would improve their wellbeing, other interventions have to be considered. One strategy to support active aging could be multimodal coaching approaches (61). For that purpose, application of digital health technology could be beneficial. Such digital coaching interventions could suggest users who were nearby green or blue spaces that can be easily accessed, or where this is not possible. In these cases, family, caregivers, and social workers are needed to help them to cope with these challenges. Further, the usage of attractive pictures or videos of landscapes and forest environment could be considered where people have no access to such places (i.e., elderly in nursing homes) (62). In Mostajeran et al.'s study, photos were more effective to reduce physiological arousal and to prevent mood disturbance than immersive 360° videos (62). Thus, there are different options that could be utilized to support people during difficult phases in their life and to improve public health in general.

## Data availability statement

The datasets presented in this article are not readily available due to data protection regulations. Requests to access the datasets should be directed to the corresponding authors.

## Ethics statement

Ethical review and approval was not required for the study on human participants in accordance with the local legislation and institutional requirements. Written informed consent for participation was not required for this study in accordance with the national legislation and the institutional requirements.

## Author contributions

AB designed the study and set up the online survey. KB contributed to the interpretation of data and writing the manuscript. Data were analyzed by AB and DR. All authors contributed to the article and approved the submitted version.

## Conflict of interest

The authors declare that the research was conducted in the absence of any commercial or financial relationships that could be construed as a potential conflict of interest.

## Publisher's note

All claims expressed in this article are solely those of the authors and do not necessarily represent those of their affiliated organizations, or those of the publisher, the editors and the reviewers. Any product that may be evaluated in this article, or claim that may be made by its manufacturer, is not guaranteed or endorsed by the publisher.
